# Persisting Mixed Cryoglobulinemia in Chikungunya Infection

**DOI:** 10.1371/journal.pntd.0000374

**Published:** 2009-02-03

**Authors:** Manuela Oliver, Marc Grandadam, Catherine Marimoutou, Christophe Rogier, Elisabeth Botelho-Nevers, Hugues Tolou, Jean-Luc Moalic, Philippe Kraemer, Marc Morillon, Jean-Jacques Morand, Pierre Jeandel, Philippe Parola, Fabrice Simon

**Affiliations:** 1 Laboratoire de Biochimie, Hôpital d'Instruction des Armées Laveran, Marseille, France; 2 Unité de Virologie Tropicale, Institut de Médecine Tropicale du Service de Santé des Armées, Marseille, France; 3 Comité Recherche, HIA Laveran, Marseille, France; 4 Service de Pathologie Infectieuse et Tropicale, Hôpital d'Instruction des Armées Laveran, Marseille, France; 5 Laboratoire de Biologie, HIA Laveran, Marseille, France; 6 Service de Dermatologie, HIA Laveran, Marseille, France; 7 Service des Maladies Infectieuses et Tropicales, Centre Hospitalo-Universitaire Nord, Assistance Publique Hôpitaux de Marseille, Marseille, France; Case Western Reserve University School of Medicine, United States of America

## Abstract

**Background:**

Chikungunya virus (CHIKV), an arbovirus, is responsible for a two-stage disabling disease, consisting of an acute febrile polyarthritis for the first 10 days, frequently followed by chronic rheumatisms, sometimes lasting for years. Up to now, the pathophysiology of the chronic stage has been elusive. Considering the existence of occasional peripheral vascular disorders and some unexpected seronegativity during the chronic stage of the disease, we hypothesized the role of cryoglobulins.

**Methods:**

From April 2005 to May 2007, all travelers with suspected CHIKV infection were prospectively recorded in our hospital department. Demographic, clinical and laboratory findings (anti-CHIKV IgM and IgG, cryoglobulin) were registered at the first consultation or hospitalization and during follow-up.

**Results:**

Among the 66 travelers with clinical suspicion of CHIKV infection, 51 presented anti-CHIKV IgM. There were 45 positive with the serological assay tested at room temperature, and six more, which first tested negative when sera were kept at 4°C until analysis, became positive after a 2-hour incubation of the sera at 37°C. Forty-eight of the 51 CHIKV-seropositive patients were screened for cryoglobulinemia; 94% were positive at least once during their follow-up. Over 90% of the CHIKV-infected patients had concomitant arthralgias and cryoglobulinemia. Cryoglobulin prevalence and level drop with time as patients recover, spontaneously or after short-term corticotherapy. In some patients cryoglobulins remained positive after 1 year.

**Conclusion:**

Prevalence of mixed cryoglobulinemia was high in CHIKV-infected travelers with long-lasting symptoms. No significant association between cryoglobulinemia and clinical manifestations could be evidenced. The exact prognostic value of cryoglobulin levels has yet to be determined. Responsibility of cryoglobulinemia was suspected in unexpected false negativity of serological assays at room temperature, leading us to recommend performing serology on pre-warmed sera.

## Author Summary

Chikungunya virus is present in tropical Africa and Asia and is transmitted by mosquito bites. The disease is characterized by fever, headache, severe joint pain and transient skin rash for about a week. Most patients experience persisting joint pain and/or stiffness for months to years. In routine practice, diagnosis is based upon serology. Since 2004 there has been an ongoing giant outbreak of Chikungunya fever in East Africa, the Indian Ocean Islands, India and East Asia. In parallel, more than 1,000 travelers were diagnosed with imported Chikungunya infection in most developed countries. Considering the clinical features of our patients (joint pain), we hypothesized that cryoglobulins could be involved in the pathophysiology of the disease as observed in chronic hepatitis C infection. Cryoglobulins, which are immunoglobulins that precipitate when temperature is below 37°C, can induce rheumatic and vascular disorders. From April 2005 through May 2007, we screened all patients with possible imported Chikungunya infection for cryoglobulins. They were present in over 90% of patients, and possibly responsible for the unexpected false negativity of serological assays. Cryoglobulin frequency and levels decreased with time in recovering patients.

## Introduction

Chikungunya fever is an emerging arboviral disease characterized by a brief fever, headache and myalgias, occasional evanescent rash, inflammatory polyarthralgias, arthritides or tenosynovitis that can last for months to years [1]–[4]. Chikungunya virus (CHIKV) was identified in the 1950s in Africa [2], and soon after in Asia [5]. It can be responsible for major epidemics, sometimes separated by silent periods [6]. From 2004 to 2006, a giant CHIKV outbreak successively swept out Kenya and most islands of western Indian Ocean [7]. In Réunion Island, the outbreak was explosive at the beginning of 2006 with a pick of 45,000 cases per week. Up to 2006 June 1^st^ , about one third of the 770,000 residents had been infected [8]. Another huge outbreak recently stroke India with 2 to 7 million estimated cases [9], and is currently spreading to Southeastern Asia [10]. During this period, Chikungunya fever was also identified in more than 1,000 travelers returning from the epidemic areas to European countries [1],[11],[12] and the USA [13],[14]. CHIKV-infected travelers included viremic patients who returned home to countries where competent vectors are present, raising serious concern for the globalization of the disease [7],[15]. The Italian outbreak in August 2007 has demonstrated the reality of this threat [16].

During the recent CHIKV outbreaks, previously described clinical features [3],[4] as well as the low rate of asymptomatic infections were confirmed [7],[17]. The disease was also responsible for unusual and unfrequent complications, including severe newborn infections after peripartum mother-to-infant transmission, meningo-encephalitis, hepatitis, myocarditis, severe epidermolysis [17],[18] and lead to surmortality [8]. Transitory peripheral vascular disorders (PVD), mostly Raynaud syndrome, were also observed few weeks after the disease onset [1]. Considering persistent arthralgias and occasional PVD, we hypothesized that cryoglobulin could be involved in the pathophysiology of the disease, as described for hepatitis C infection. In this infection high level of mixed cryoglobulinemia is commonly detected in patients with chronic viral replication and is strongly associated with liver damage and peripheral neuropathies [19],[20].

## Methods

### Patients

From April 2005 throughout May 2007, all patients with possible imported CHIKV infection (recent travel in Indian Ocean islands and presence or history of fever and/or arthralgias) were prospectively recorded at Laveran Military Hospital in Marseille, France. The criteria for confirmed cases were i) presence of specific anti-CHIKV IgM and/or positive RT-PCR and/or isolation of CHIKV from blood and/or specific anti-CHIKV IgG, ii) recent clinical feature consistent with CHIKV infection, iii) no other etiology identified. Demographic, clinical and laboratory findings were registered for all patients at their first consultation or hospitalization and during follow-up. For patients seen more than 10 days after the onset of illness, early clinical features were identified using a retrospective questionnaire. The clinical status of each patient was actively monitored by the same physician (FS) during consultations and/or by phone calls every 2–3 months. The early stage is defined as the first 10 days of clinical disease while second stage is defined as symptoms and signs persisting more than 10 days after disease onset [1].

The same physician (FS) orally informed all patients about the requirement of blood samples to diagnose the aetiology of their polyarthralgia. The script for obtaining oral consent was accepted by the Institutional Review Board at Laveran Hospital. All patients gave their oral consent, which was noted in their individual medical file. A single blood sample of less than 50 cc was taken after consultation, when clinically required; no supplementary blood samples were taken for research.

### Laboratory Procedures

At each consultation, patients were tested for CHIKV serology using in-house IgM-capture and IgG-sandwich enzyme-linked immunosorbent assays [6]. Serologies were performed first on sera kept at 4°C before analysis and second, on sera kept at 37°C until analysis. IgG and IgM for West Nile, dengue and Rift Valley fever viruses were also assayed. On acute sera, RT-PCR and isolation of CHIKV in Vero-E6 cells were attempted [21]. Cryoglobulinemia was screened using the following procedure: i) collection of 14 ml of blood in pre-warmed tubes at the hospital's biochemistry laboratory, ii) clotting at 37°C for 3 hours, iii) centrifugation at 37°C (3,000 RPM for 10 minutes), iiii) freezing of the pellet at 4°C for 10 days, to allow generation of cryoprecipitates. Purification, characterization and quantification of cryoglobulins were performed by the same operator (MO), as previously described [22]. Cryoglobulins were classified as type I for monoclonal component, type II for one monoclonal component associated with polyclonal immunoglobulins, type II–III for more than one monoclonal component associated with polyclonal immunoglobulins, and type III for polyclonal immunoglobulins [23],[24]. Type III cryoglobulinemia was considered positive when level was higher than 5 mg/L [22], whereas type II and type II–III cryoglobulins were considered positive regardless of concentration level [22]. CHIKV RNA in cryoprecipitates was searched using RT-PCR, as described elsewhere [21].

Patients also underwent immunological tests: (i) determination of C3 and C4 complement components using an immunonephelemetric method (Dade Behring Paris France), (ii) determination of total haemolytic complement using the total haemolytic complement kit (The Binding Site Saint Egreve France), (ii) search for rheumatoid factors, by latex immunoagglutination (Biomérieux Marcy l'Etoile France), (iv) detection of antinuclear antibodies by indirect immunofluorescence (Eurobio Paris France). Serological screening for hepatitis C virus (HCV) infection was systematically performed using sandwich enzyme-linked immunosorbent assays (Biorad Marne La Coquette France). After 6 months, blood testing for asymptomatic patients was restricted to CHIKV serology and detection of cryoglobulinemia.

### Statistical Analysis

Chi 2, Kruskall-Wallis and exact Fisher's, two-tailed probability tests were used with a significant p value of 0.05 (Stata 9.0 Software, StataCorp College Station, Texas, USA).

## Results

### Patients and First Screening for CHIKV Infection

During the 25 month-study, 66 French patients with clinical suspicion of CHIKV infection were prospectively included. Among them, 51 presented with anti-CHIKV IgM *(see below)*. Fifteen patients remained seronegative for both anti-CHIKV IgM and IgG. No patients had detectable CHIK virus in sera (RT PCR and CHIK V isolation on Vero cell were negative).

Sex ratio (M/F) and median age in seropositive patients were respectively 1.04 and 54 years (range: 21 y–78 y), *versus* 0.67 and 47 years (21 y–75 y) in seronegative patients (no statistical difference). Six CHIKV-infected patients were lost to follow-up after the first consultation. The 45 other patients have been followed in our medical unit for a median duration of 14 months (range: 54 days–25 months), through 2 to 6 consultations per patient; resulting in a total of 138 consultations for CHIKV patients performed up to May 2007.

Table 1 summarizes the demographic, epidemiological and clinical events of the 51 confirmed cases. Ninety-eight percent of the seropositive cases suffered at least once with arthralgia, 71% with tenosynovitis and 20% with transitory PVD. Thirteen patients experienced at least one clinical relapse during follow-up, *i.e.* became symptomatic again after at least one symptom free month, mostly with subacute arthralgias in hands and feet. Thirteen patients developed *de novo* transitory PVD, mainly in fingers and sometimes in toes, within the second and third months after the disease onset. No other aetiology (drug, auto-immune disorder, Buerger disease, local trauma) than CHIKV was identified for relapses or PVD. No morphological changes were observed in the 3 patients for whom a digital capillaroscopy was performed. PVD was not significantly associated with gender. Conversely, tenosynovitis was significantly more frequent in women, both on the day of consultation (p = 0.01) or during the previous month (p = 0.04).

**Table 1:** Characteristics of 51 patients with Chikungunya infection imported from Indian Ocean Islands between April 2005 and October 2006, to Laveran Military hospital, Marseille, France.

***Demographic and epidemiological data***	
Median age (ranges)	54.0 years (21–78 y)
Sex ratio M/F	1.04 (26/25)
Chronic general or rheumatic disease	37
Year of contamination	
2005	12
2006	39
Location of contamination	
Reunion	32
Comoros archipelago	9
Mauritius	4
Madagascar	3
Seychelles	1
Reunion or Mauritius	1
Location of disease onset	
Western Indian Ocean	34
Mainland France	17
Mean duration of stay	
Residents in Western Indian Ocean	9 days
No residents	54 days
Hospitalization	
During early (acute) stage	4
During chronic stage	8
***Acute stage (within the first 10 days)***	
Fever (mean duration)	47 (3.5 days)
Mucous manifestations	
Gingival or nasal bleeding	3
Conjunctivitis	3
Mouth ulcers	2
Peripheral arthralgias/ arthritis	50
Number of involved joint groups	51
None	1
Less than 10	20
Ten or more	30
Hands and/or feet involvement	48
Symmetry of joint involvement	44
Periarticular oedema	20
Axial involvement	29
Tenosynovitis	17
Rash	21
Pruritis	10
Nervous tunnel syndrome	3
Peripheral vascular disorder	3 (finger coldness)
Complication	1 (acute myocarditis)
***Chronic stage (after the 10^th^ day)***	
Chronic joint pain and/or stiffness	48
Hand and/or feet involvement	47
Symmetry of joint involvement	43
Exacerbation of pre-existing pains	14
Axial involvement	21
Tenosynovitis/tendonitis/tendon rupture	34
Nervous tunnel syndrome	17
Peripheral vascular disorder	
Raynaud syndrome	5
Erythermalgia	3
Finger coldness	4
Pulpar necrosis	1
Short-term general corticotherapy	26
Once	14
Twice	8
Three times	3
Four times	1

This table reports the most frequent signs and symptoms, regardless of duration.

Clinical characteristics of 39 of these patients have been described elsewhere [1].

### Cryglobulinemia Testing

Among the 51 CHIKV-infected patients, cryoglobulinemia was screened for 48, *i.e.* 42 patients on first consultation and 6 later during follow-up. Cryoglobulinemia screening was repeated 2 to 5 times for 38 patients during the study period. The median delay between CHIKV acute symptomatology onset and first cryoglobulinemia testing was 45 days (range: 5–640 d). On first consultation, 37/42 patients (88%) were cryoglobulinemic. Among the five patients without cryoglobulinemia: i) three symptomatic patients respectively free of cryoglobulinemia on days 5, 16 and 36 after disease onset, developed cryoglobulinemia on days 27, 45 and 69, respectively; ii) one symptomatic patient free of cryoglobulinemia on his first test at day 124 (a week after a short-term corticotherapy) became positive with a type II on day 204; iii) one patient had been symptom-free for weeks and was not cryoglobulinemic when tested on day 107 after disease onset.

Among the 6 patients first tested at the second consultation, 4 were cryoglobulinemic and two remained negative for cryoglobulin on day 143 and day 355, respectively, while they were both symptom-free. Finally, 94% of the 48 CHIKV-infected tested patients were positive for cryoglobulinemia at least once during their follow-up.

During the study period, 118 cryoglobulin assays were performed, 83 were positive. There were 61% (51/83) type II, 30% (25/83) type II–III and 8% (7/83) type III. Monoclonal IgM-κ and IgM-λ components were found in 56/83 cryoglobulins (68%) and 48/83 (58%), respectively; polyclonal IgM components in 83/83 cryoglobulins (100%). Monoclonal IgA was found in only 2/83 cryoglobulins. Thus, all cryoglobulins associated with CHIKV infection were mixed cryoglobulins.

The cryoglobulin concentration could be determined for 79/83 cryoglobulinemias. The median cryoglobulin level was 8 mg.L^−1^ (range: 3–165). No CHIKV was detected by RT-PCR in 24 tested cryoprecipitates.

#### Cryoglobulinemia and serological assay for CHIKV infection

When performed on sera kept at 4°C before analysis, detection of anti-CHIKV IgM was positive for 45 /66 patients. Among them, cryoglobulinemia was found in 40 patients (89%). Moreover, cryoglobulins were detected in 11 (52%) of the 21 initially seronegative patients. For these 11 patients new ELISA were conducted on sera kept at 37°C until analysis, to allow dissolution of putative cryoprecipitates. Anti-CHIKV IgM were found in 6 of these patients and anti CHIKV IgG in 4. Pre-warmed sera were characterized by high levels of specific antibodies and presence of cryoglobulin in 5 /6 cases. When summarizing, a total of 51 patients were serologically confirmed with CHIKV infection. Ninety-four percent where cryoglobulinemic at least once during follow up, while only 27% (4/15) of CHIKV-seronegative patients were cryoglobulinemic (p<0.0001). All 51 patients but one (98%) were positive for anti-CHIKV IgM and 88% for IgG. The remaining patient, unless presenting with typical CHIKV-clinical symptoms, became anti-CHIKV IgG positive only when tested 124 days after disease onset.

#### CHIKV-associated cryoglobulinemia, clinical features and corticotherapy

**Figure 1:**
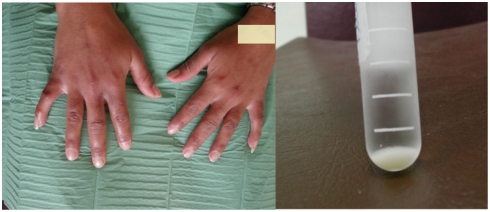
Left side: Red stage of Raynaud syndrome and swelling of finger joints in a 36-year-old CHIKV-infected woman 6 weeks after disease onset; right side: concomitant cryoprecipitate in her blood.

There was a negative correlation between age and cryoglobulin levels (p = 0.012). As arthralgias and cryoglobulinemia were quasi constant (prevalence 98% and 94%, respectively) no statistical association could be tested. No significant difference was found in cryoglobulin type or concentration according to presence of tenosynovitis or number of joints involved (less or more than 10). Although patients with PVD presented with more type II–III cryoglobulins (25%) than patients without PVD (13%), the difference was not significant. Nevertheless a cryoglobulin concentration higher than 8 mg.L^−1^ (median concentration) was present in 7/31 samples from patients suffering with PVD *versus 2*/39 samples from patients without PVD (*p* = 0.06). Two specific clinical cases are particularly illustrative for pathophysiology: (i) the type II–III cryoglobulin level of a 36-year-old woman rose from 32 mg.L^−1^ on day 40 to 104 mg.L^−1^ on day 60, while she was just developing a severe Raynaud syndrome *(* Figure 1 *)*; (ii) a 20-year-old woman, whose severe acute stage was marked by transitory myocarditis and finger coldness, also presented high levels of type III cryoglobulin, up to 165 mg.L^−1^ on day 85. No other specific clinical sign was presented by patients whose cryoglobulin level was below 100 mg.L^−1^.

Twenty-six patients received short-term general corticotherapy, mainly prescribed for disabling distal polyarthritis, multiple tenosynovitis or severe PVD, when non-steroidal anti-inflammatory drugs (NSAIDs) were contraindicated (4 cases) or ineffective (22 cases). All of them experienced definitive or transitory improvement of tendon pains, PVD and sometimes arthralgias after this treatment.

The parallel evolution of symptoms and cryoglobulinemia under corticotherapy in a 54-year-old CHIKV-infected woman was particularly illustrative. On day 34 after disease onset, she was disabled by severe polyarthritis and tenosynovitis and a type II cryoglobulin was identified at 15.2 mg.L^−1^. As NSAIDs were contraindicated because of oral anticoagulation, she received general corticotherapy with progressive withdrawal within the 3^rd^ month. At the end of this treatment, her clinical status improved dramatically while cryoglobulinemia disappeared. After 8 months of evolution, a low level (4.06 mg.L^−1^) of type II–III cryoglobulin was detected, while she only complained of mild stiffness in the fingers. This case was suggestive for an unexpected role of MC in pathophysiology of late CHIKV manifestations.

#### Evolution Of CHIKV-associated cryoglobulinemia

**Figure 2:**
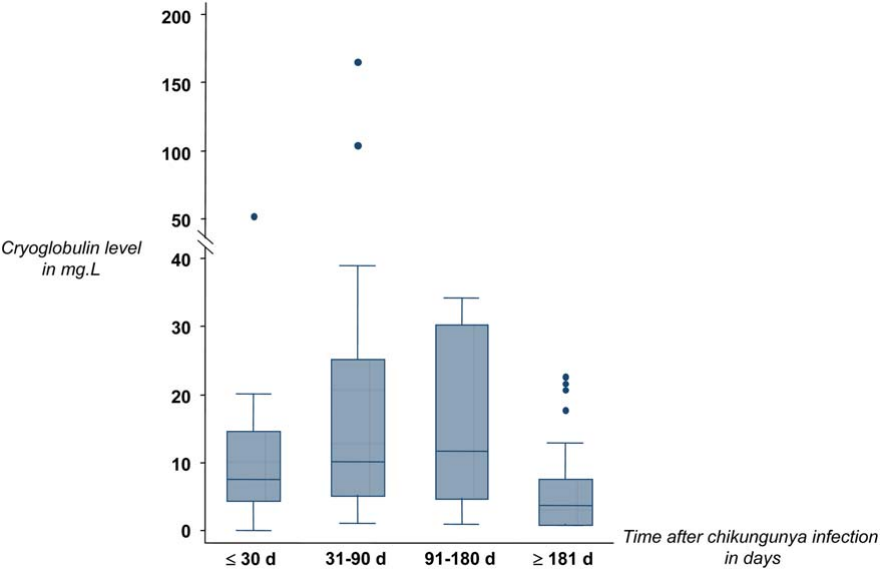
Evolution of all types cryoglobulin levels over time in 51 Chikungunya-infected travelers, Laveran Military Hospital, Marseille, France. The sample results are presented as follows: box represents the interquartile range, the included line figuring the median; lines out of the box represent range with extreme outliers figured as points.

Cryoglobulin was screened in 39 patients throughout the follow-up period. The earliest cryoglobulins were detected on day 6 after disease onset. Cryoglobulin prevalence rate was negatively associated with elapsed time after disease onset (p = 0.002). Prevalence remained stable around 80% within the first three months, and decreased to 57% in the second semester (*Table 2*). Cryoglobulins were still detected in 55% of the 18 patients evaluating for more than a year. Types II and III cryoglobulins seemed to be detected earlier than type II–III cryoglobulins (median delay: 36.5 and 41 days *versus* 58.5 days, respectively; p = 0.07). The evolution of cryoglobulin levels over time after CHIKV infection onset is presented in Figure 2. Type II and II–III cryoglobulin levels significantly changed over time (type II, p = 0.008; type II–III, p = 0.04; *table 2*). Interestingly, a type II cryoglobulin at 6 mg.L^−1^ was detected in a Comorian woman who was still painful one year after her infection in the Comoros archipelago. On day 596, no cryoglobulin was detected, whereas the patient had become asymptomatic for 4 months.

**Table 2:** Cryoglobulin types and levels regarding delay after Chikungunya infection onset, at Laveran Military hospital, Marseille, France.

Delay	No cryoglobulin Prevalence *(%)*	Type II^a^ Prevalence *(%)* [median level]	Type II–III^b^ Prevalence *(%)* [median level]	Type III Prevalence *(%)* [median level]	All types^c^ Prevalence *(%)* [median level]
0–30 days	2/16 *(12.5%)*	10/16 *(62.5%)* [14.5 mg.L^−1^]	2/16 *(12.5%)* [2.7 mg.L^−1^]	2/16 *(12.5%)* [6.8 mg.L^−1^]	14/16 *(87.5%)* [7.7 mg.L^−1^]
31–90 days	3/28 *(10.7%)*	14/28 *(50.0%)* [10.1 mg.L^−1^]	7/28 *(25.0%)* [25.2 mg.L^−1^]	4/28 (14.3%) [35.9 mg.L^−1^]	25/28 (89.3%) [10.4 mg.L^−1^]
91–180 days	10/27 *(37.0%)*	10/27 *(37.0%)* [6.54 mg.L^−1^]	7/27 *(25.9%)* [26.2 mg.L^−1^]	0/27 *(0%)*	17/27 *(63.0%)* [11.7 mg.L^−1^]
181–747 days	20/47 *(42.6%)*	17/47 *(36.2%)* [3.1 mg.L^−1^]	9/47 *(19.1%)* [4.1 mg.L^−1^]	1/47 *(2.1%)* [21.6 mg.L^−1^]	27/47 *(57.4%)* [3.7 mg.L^−1^]

a p = 0.008.

b p = 0.04.

c p = 0.002 (Kruskall-Wallis test for distribution between the different delays).

**Figure 3:**
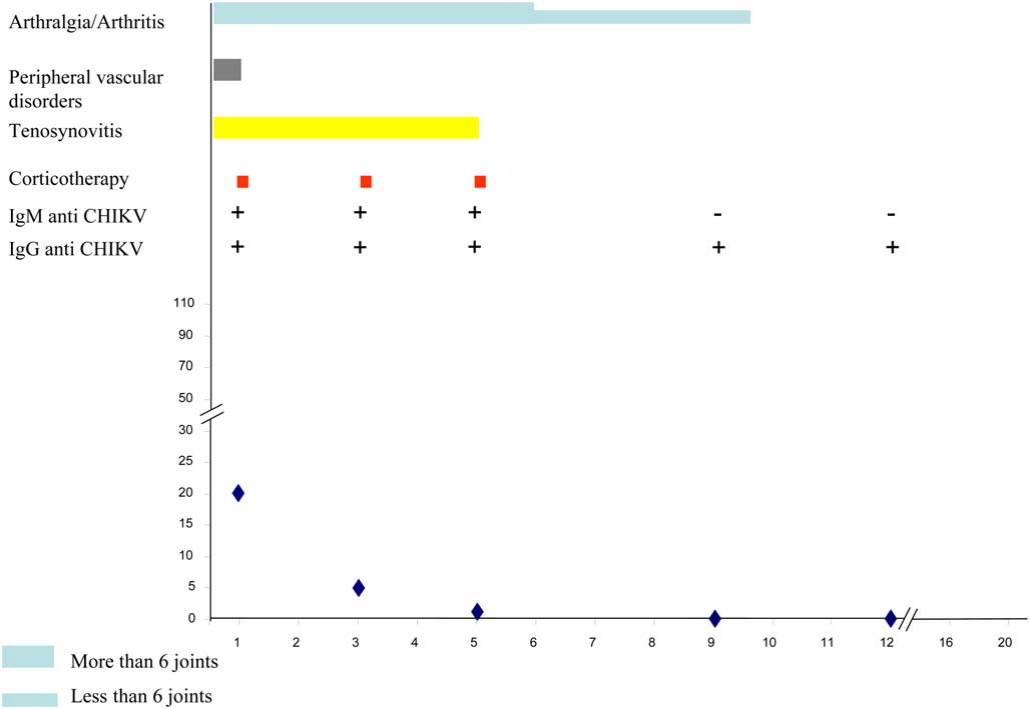
Individual follow up of a 62-year-old female patient with CHIKV infection. X-axis, month of follow-up; Y-axis, Cryoglobulin level in mg.L-1.

**Figure 4:**
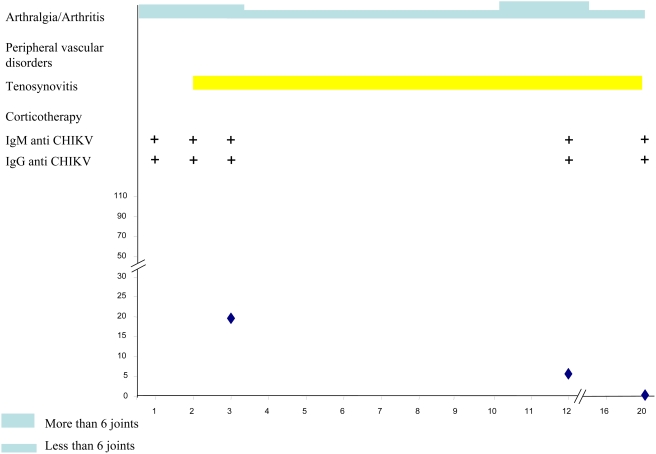
Individual follow up of a 41-year-old female patient with CHIKV infection. X-axis, month of follow-up; Y-axis, Cryoglobulin level in mg.L-1.

**Figure 5:**
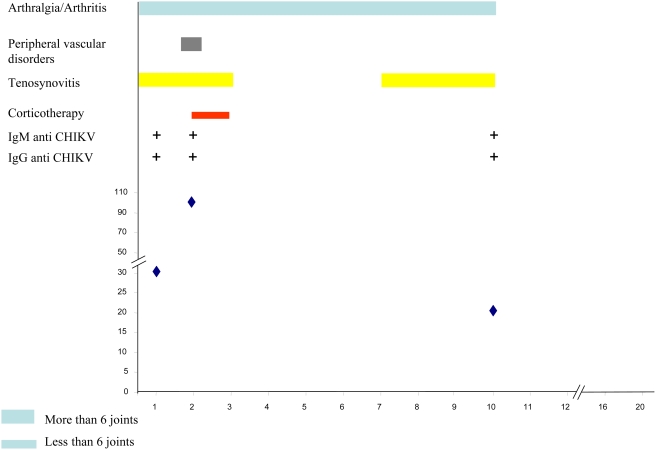
Individual follow up of a 36-year-old female patient with CHIKV infection. X-axis, month of follow-up; Y-axis, Cryoglobulin level in mg.L-1.

**Figure 6:**
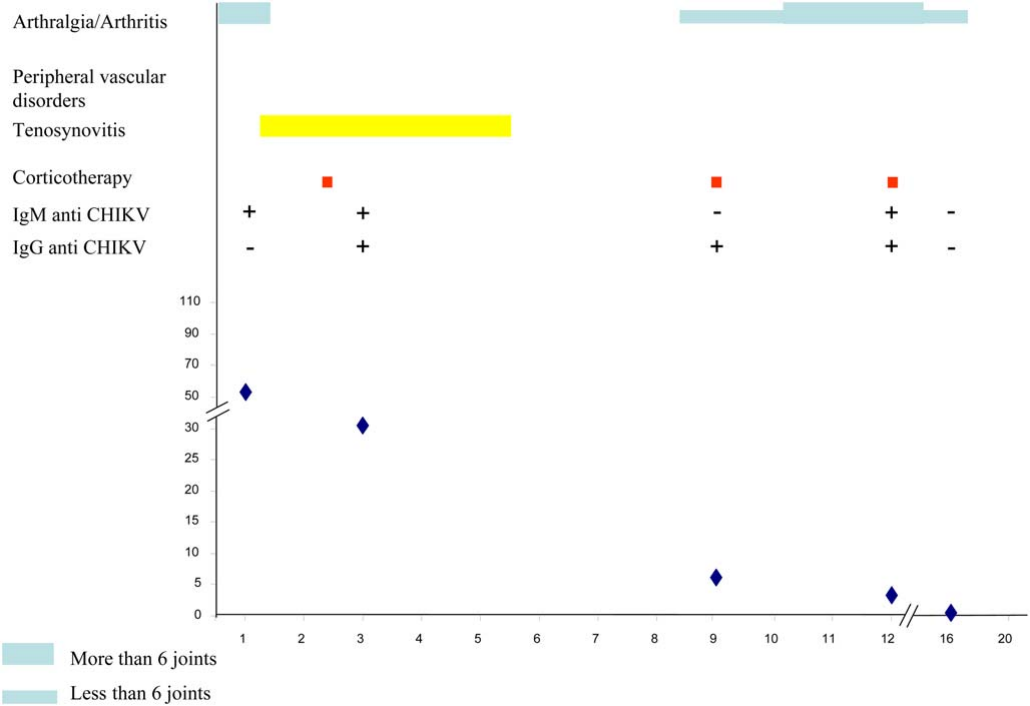
Individual follow up of a 62-year-old male patient with CHIKV infection. X-axis, month of follow-up; Y-axis, Cryoglobulin level in mg.L-1.

**Figure 7:**
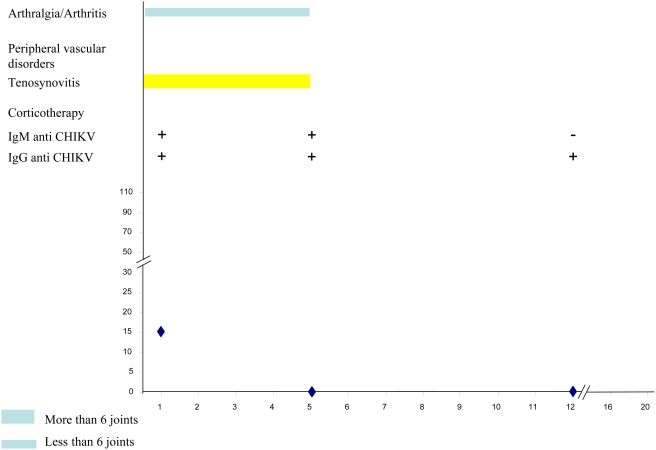
Individual follow up of a 66-year-old female patient with CHIKV infection. X-axis, month of follow-up; Y-axis, Cryoglobulin level in mg.L-1.

Table 3 summarizes the main clinical and biological data of the CHIKV-infected patients at successive times during follow-up. Figures 3, 4, 5, 6 and 7 illustrate examples of clinical and biological evolution after CHIKV infection.

**Table 3:** Clinical and biological status of 51 patients with Chikungunya infection at successive follow-up delays, Laveran Military hospital, Marseille, France (M: month).

	M1	M2	M3	M5	M7	M10	M13
**Patients with available status**	51	50	47	47	46	44	31
**Symptomatic patients**	50	47	43	38	32	28	18
**Cryoglobulemic/all tested patients**	13/15	16/17	11/13	10/15	11/18	9/13	10/19^a^
**Cryoglobulinemic patients/symptomatic tested patients**	12/14	16/17	10/12	8/11	8/13	5/9	8/13
**Cryoglobulinemic patients/asymptomatic tested patients**	1/1	-	1/1	2/4	3/6	4/4	2/6
**Patients with anti-CHIKV IgM /all tested patients**	18/18	17/17	12/12	12/15	12/16	5/12	2/18
**Patients with both anti-CHIKV IgM & cryoglobulinemia/tested patients**	13/15	16/17	11/12	9/15	7/16	5/12	2/18^b^
**Patients with anti-CHIKV IgG/tested patients**	12/18	16/17	11/12	14/15	14/16	10/12	13/18

a one patient underwent two cryoglobulin tests, the one performed one week after general corticotherapy is not presented here.

b these two patients remained symptomatic even after 15 months.

### Other Laboratory Testing

Neither antinuclear antibody nor rheumatoid factors were found during follow-up. C3, C4 complement fractions and haemolytic complement levels remained normal. All patients were seronegative for HCV.

## Discussion

Our study was an empiric prospective study of symptomatic CHIKV-infected travelers coming back from Western Indian Ocean. Thus, follow-up for cryoglobulinemia was not performed in seronegative patients in the absence of ethic committee consent.

Up to now, pathogenesis of CHIKV-induced rheumatism remains unknown. Fourie *et al.* identified low titers of rheumatoid factor in CHIKV infection [3], whereas no antinuclear antibodies or rheumatoid factors were detected here like in previous studies [4]. We identified for the first time the association between CHIKV infection and cryoglobulinemia among infected travelers. As no type I cryoglobulin (composed of one monoclonal immunoglobulin) was detected in our patients, CHIKV seems to induce only mixed cryoglobulinemia (MC), either type II, II–III or III.

MC has already been described in miscellaneous acute and chronic infections [19],[20]. In these cryoglobulinemia seemed to rapidly decrease and disappear within a few weeks of pathogen clearance, although persisting MC has been observed in few chronic infections, the most common being chronic hepatitis C virus (HCV) (MC prevalence range: 20–55%) [19],[20]. In our study, the strength of association between CHIKV infection and MC was much higher 94%. A biased high prevalence of CHIKV-MC due to use of a highly sensitive method can be ruled in regard of the frequent negativity of cryoglobulinemia detection over time during follow-up.

The persistence of CHIKV-associated MC (CHIKV-MC) was unexpected, as the CHIKV genome has never been detected in blood after 12 days of disease evolution, even in chronic symptomatic patients [25]. Consistently, we failed to detect CHIKV-RNA in the cryoprecipitates, as described in chronic HCV infection [26],[27]. The absence of chronic viremia could also explain the much lower level of CHIKV-MC than those observed in chronic HCV infection [22]. Ozden *et al.* recently showed the presence of CHIKV in muscle satellite cells of a non-immunocompromised patient who was still painful three months after disease onset [28]. Moreover, Jaffar-Bandjee *et al.*, using molecular tools, evidenced persistence of CHIKV in perivascular macrophages of synovial tissue in a chronic elbow hygroma of a patient infected for one year [29]. This discovery suggests that CHIKV can induce a chronic infection with active replication, conversely to most other arboviruses. Persistence of CHIKV also seemed to stimulate host immunity, as inflammation with macrophages and T cells was locally concomitantly observed. Thus, viral replication could be involved in the prolonged persistence of anti-CHIKV IgM and, as a consequence, of CHIKV-MC in patients with long-persisting symptoms. CHIKV-MC should be the possible “missing link” between CHIKV and some symptoms of the chronic stage.

The second main finding is the existence of false seronegativity in one out of three and a half CHIKV-infected patient with typical clinical presentation of CHIKV infection when using “classical” ELISA assay at room temperature. This phenomenon can be responsible for non-recognition of CHIKV infection in individuals with chronic rheumatism, leading to useless explorations, as well as underestimation of seroprevalence in an endemic/epidemic area. Considering the high prevalence of CHIKV-MC in our cohort, we assume that cryoglobulinemia may be a significant factor in misdiagnosing the disease when using “conventional” serology. Specific anti-CHIKV antibodies, *i.e.* IgM and IgG, could all be trapped in the cryoprecipitate, as already described in chronic HCV infection [30]. Therefore, when facing a patient with a clinical suspicion of CHIKV infection and with paradoxical seronegativity, we recommend the following procedure: at least pre-warmed the serum at 37°C before serology testing or, preferably, manage the blood sample as required for any cryoglobulin research: sampling and centrifugation at 37°C, decantation and serum pre-warming before the ELISA assays. However, considering the high prevalence of MC among our CHIKV-seronegative patients (40%) and the unexpected persistent seronegativity in a few patients with a high clinical suspicion of CHIKV-infection, we cannot exclude some residual false negative results due to the precipitation of CHIKV-MC before blood centrifugation.

Chronic MC is commonly associated with various symptoms, mostly arthralgias, purpura, weakness and Raynaud syndrome [19],[20]. In our study, no CHIKV-infected patient presented the complete clinical triad associated with MC, but arthralgias were present in all and combined with PVD in 24%. The concomitancy of arthralgias and cryoglobulinemia in more than 90% of the cases is consistent, although not demonstrative, with an involvement of cryoglobulin in arthralgias. Frequent exacerbation of rheumatic pain and handicap, and the occasional incidence of transitory PVD have been recently described within the 2^nd^ and/or 3^rd^ months after CHIKV-infection onset [1]. These clinical manifestations are synchronous with increasing cryoglobulin levels. The low cryoglobulin blood levels -when compared with chronic HCV infection- could explain the lack of vascular purpura in our cohort. No significant association between PVD and CHIKV-MC prevalence was observed, possibly due to the small number of patients, although there was a positive trend between presence of PVD and CHIKV-MC levels (p = 0.06). We failed to find any significant association between CHIKV-MC type or level and any other sign or symptom.

Most patients self-declared improvements after short-term general corticotherapy. In few of them we could evidence concomitant CHIKV-MC disappearance. Corticosteroids could interfere through two actions: immunosuppression of B lymphocyte activation and/or decrease of joint and tendon inflammation. Randomized studies are needed to confirm the benefits and risks of general corticotherapy and to specify therapeutic modalities in chronic CHIKV-associated rheumatism. Complementary investigations are also required to determine whether antiviral drugs, such as chloroquine or interferon could be helpful in stopping CHIKV replication in muscle satellite cells and MC production in symptomatic chronic patients, as suggested elsewhere [31].

Finally, the present work also shows the evolution of CHIKV-MC types and levels over time. Schematically, type III or type II cryoglobulin appear first (median delay: around 40 days), followed by type II–III (median delay: around 2 months), conversely to what is usually described in HCV infection [32]. Cryoglobulin levels reached their acme within the 3rd month after disease onset and remained stable up to the 6th, before a slow decline. Regarding the parallel evolution of CHIKV-MC and anti-CHIKV IgM antibodies over time and the systematic detection of IgM in CHIKV-MC, the direct involvement of these antibodies in cryoprecipitates can be suspected. After 6 months of evolution, CHIKV-MC levels decrease for most patients, in parallel with obvious clinical improvement. No cryoglobulin was detected in most of patients after they became symptom-free, whatever the delay of recovery. However the evolution was not linear. About one quarter of the patients underwent clinical relapses with distal arthralgias and a concomitant increase or reappearance of cryoglobulin. The disappearance of CHIKV-MC could be predictive for a clinical cure in chronic CHIKV disease, as described in HCV infection after interferon and ribavirin or rituximab treatment [19]. Further studies with long time follow-up are required to determine if type or level of early CHIKV-MC has a real prognostic value. In Brighton's experience, 87.9% of 107 CHIKV-infected patients self-declared cured 3 years after disease onset, while 12.1% mentioned persistent symptoms including occasional discomfort, persistent joint stiffness, or stiffness and pain and effusion (3.7%, 2.8%, and 6% respectively) [4].

CHIKV infection is currently spreading in Africa, Indian Ocean, India and Southeastern Asia and threats many others areas where *Aedes spp.* is present. It is responsible for long-persisting symptoms, which severely impair quality of life of CHIKV-infected patients although natives or travelers. Thus, the identification of very high prevalence of CHIKV-MC is of importance. First, its presence can induce false negativity in serology performed at room temperature; leading to the recommendation of using pre-warmed sera for serology both for individual diagnosis, and seroprevalence estimation in endemic areas. Second, CHIKV-MC may be involved in the pathogenesis of the chronic stage, mainly CHIKV-associated chronic rheumatism and PVD. Third, its prolonged disappearance could be a marker of the definitive clinical cure.
